# Targeting PKM2 in cancer therapeutics: mechanistic advances and translational opportunities

**DOI:** 10.3389/fimmu.2025.1649488

**Published:** 2025-10-15

**Authors:** Lin Liu, Junyi Wang, Libo Liang, Chunhua Chen, Meng Xie, Wencheng Tang

**Affiliations:** Drug Dispending Department, The Third Hospital of Mianyang, Sichuan Mental Health Center, Mianyang, China

**Keywords:** M2-type pyruvate kinase, aerobic glycolysis, PKM2-targeted therapeutics, signaling networks, anti-cancer

## Abstract

M2-type pyruvate kinase (PKM2) serves as the key rate-limiting enzyme in aerobic glycolysis within tumor cells, where its aberrantly high expression in numerous human malignancies facilitates tumor progression by enhancing glycolytic flux through diverse signaling pathways. Beyond its metabolic function, extensive studies have established PKM2 as a critical non-metabolic signaling regulator implicated in multiple oncogenic processes, including tumor proliferation, invasion, migration, immune evasion, and resistance to chemotherapy. The elucidation of PKM2-mediated oncogenic pathways has spurred the development of targeted therapeutic strategies, positioning PKM2 as a promising target in cancer therapy. However, comprehensive reviews addressing the relationship between PKM2 and tumorigenesis remain limited. This review systematically examines the biological functions of PKM2, the signaling mechanisms through which it exerts its effects in malignant tumors, and the latest advances in the development of PKM2-targeted therapeutics, offering insights into potential directions for future drug discovery.

## 1 Introduction

The Warburg effect represents a fundamental metabolic distinction between tumor cells and normal cells, wherein tumor cells preferentially rely on glycolysis for energy production, irrespective of oxygen availability. This metabolic adaptation is characterized by excessive glucose consumption, leading to the rapid generation of lactate and ATP (1). Consequently, cancer cells exhibit a heightened capacity for glucose uptake, sustaining their uncontrolled proliferation. Furthermore, numerous intermediate metabolites derived from aerobic glycolysis facilitate the activation of alternative metabolic pathways, such as the pentose phosphate pathway, thereby supporting biosynthetic processes essential for tumor growth (2). The accumulation and extracellular secretion of metabolic byproducts, particularly lactate, contribute to the acidification of the tumor microenvironment, which in turn enhances tumor invasiveness and metastatic potential.

During aerobic glycolysis, the final irreversible step is catalyzed by pyruvate kinase (PK), a key rate-limiting enzyme responsible for pyruvate generation. PK exists in four isoforms—PKM1, PKM2, PKL, and PKR—encoded by two genes: PKLR, which encodes the L and R isoforms, and PKM, responsible for the M1 and M2 isoforms (3, 4). At the protein expression level, multiple isoforms can coexist within the same tissue. PKM1 is predominantly expressed in highly differentiated tissues such as skeletal muscle, heart, and brain, whereas PKR is exclusive to erythrocytes, PKL is specific to the liver, and PKM2 serves as the predominant isoform in the kidney (5). PKM2 is also present in various adult tissues, including the lung, white and brown adipose tissue, intestine, ovary, testis, and pancreatic islets (5). Notably, during tumorigenesis, PKM2 is abundantly expressed, progressively replacing the tissue-specific PK isoform until it becomes the dominant form within malignant cells (6).

PKM2 exists primarily in two conformational states: tetrameric form and dimeric form (7). Emerging evidence suggests that PKM2 plays a critical role not only in tumor metabolism but also in non-metabolic processes across various diseases, including cardiovascular disorders (8). Beyond its established role in cancer metabolism, PKM2 contributes to tumor progression by regulating non-metabolic pathways implicated in key metastatic processes, such as cell migration, angiogenesis, and stemness maintenance (9). Notably, PKM2 has been shown to facilitate tumor-derived exosome secretion, thereby driving cancer progression (10). Additionally, nuclear translocation of PKM2 enhances the immunosuppressive properties of tumors, further promoting hepatocellular carcinoma metastasis (11). Moreover, the latest research also indicates that Nuclear PKM2 also functions as a non-classical RNA-binding protein (RBP), competitively blocking the binding of the inhibitory RBP (HNRNPF) to the folded G-quardruplex (rG4) structures within precursor messenger RNA (pre-mRNA). This promotes the expression of pre-mRNA containing rG4, with rG4 abundance exhibiting a negative correlation with cancer patient survival rates (12). Despite the growing body of research on PKM2’s oncogenic functions, comprehensive reviews integrating its metabolic and non-metabolic roles with recent advances in PKM2-targeted therapeutics remain scarce. This review provides a systematic analysis of PKM2’s biological functions, the signaling mechanisms through which it drives tumorigenesis, and recent advancements in the development of PKM2-targeted therapies.

## 2 PKM2 expression in tumors

PKM2 has been extensively documented as being aberrantly overexpressed across various malignancies. In comparative analyses of human brain tumor samples, PKM2 was found to be significantly upregulated in glioma tissues, particularly in glioblastoma (GBM) (13). Notably, one of the defining characteristics of GBM is its altered metabolic profile, marked by a substantial increase in glycolysis, with clinical data indicating a pronounced elevation in PKM2 expression among patients with GBM (14).

**Figure 1:**
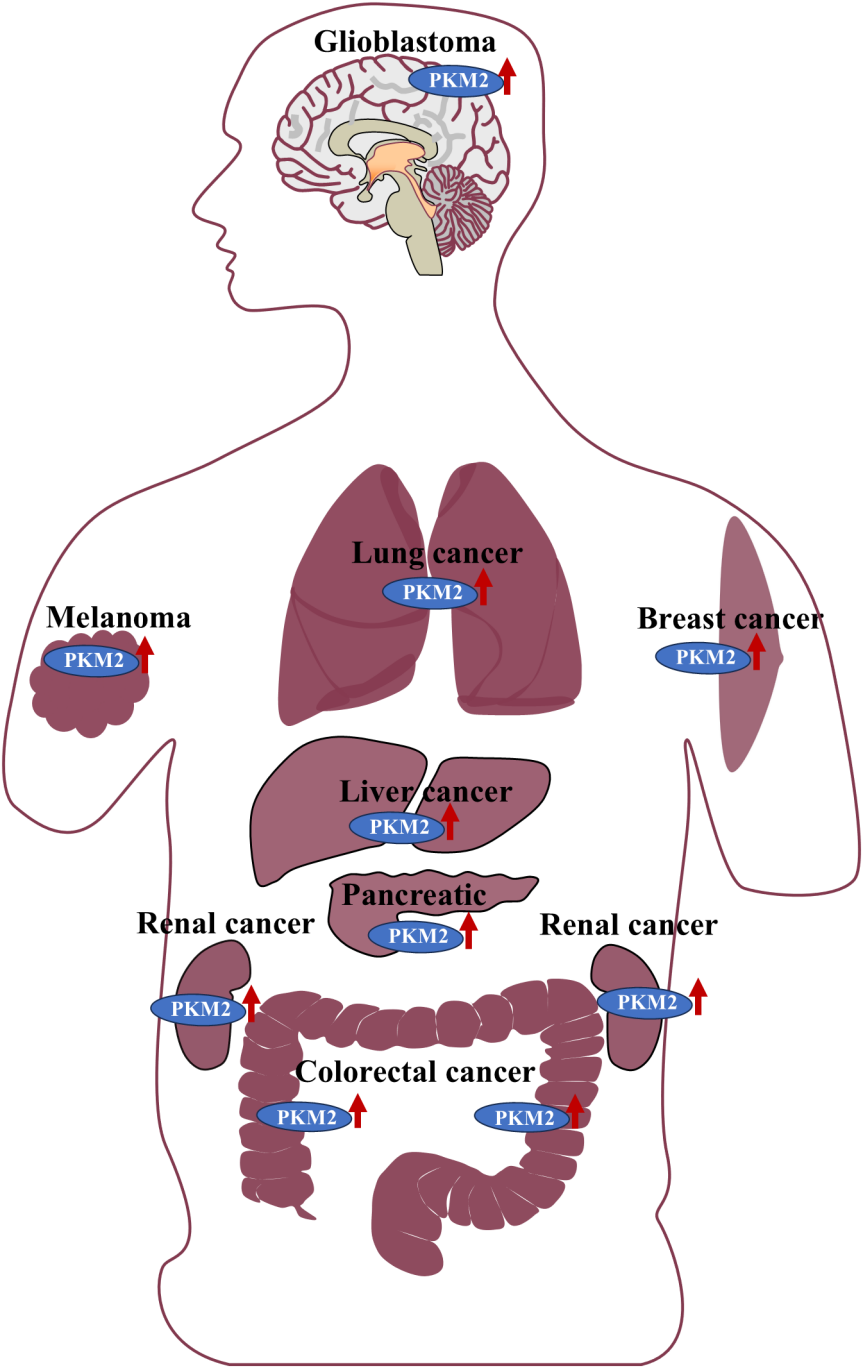
PKM2 is highly expressed in the tissues of various malignant tumors, including gliomas, lung cancer, breast cancer, melanoma, liver cancer, pancreatic cancer, kidney-related tumors, and colorectal cancer.

Mass spectrometry-based analyses of lung cancer cells and clinical specimens have also revealed high PKM2 expression and secretion, suggesting its potential as a serum biomarker for lung cancer diagnosis (15). Similarly, clinical tissue samples from lung adenocarcinoma exhibited elevated PKM2 expression, which was significantly correlated with lymph node metastasis and advanced TNM stage (16). In breast cancer, PKM2 was strongly expressed in a large cohort of clinical tissue samples, where its aberrant overexpression was linked to poor prognosis and resistance to neoadjuvant chemotherapy (17). In the highly aggressive triple-negative breast cancer (TNBC) subtype, PKM2 was found to be dysregulated and actively involved in accelerating tumor progression (18). Furthermore, PKM2 was markedly upregulated in hepatocellular carcinoma (HCC), where its expression was associated with unfavorable patient prognosis (19). In colorectal cancer (CRC), clinical tissue samples demonstrated elevated PKM2 levels, with a strong positive correlation between its expression and both lymph node metastasis and tumor stage (20). Similarly, significantly increased PKM2 expression was observed in renal cancer tissues (21). In patients with melanoma, PKM2 was highly expressed, and its activity was positively correlated with tumor malignancy and glycolytic capacity (22). Additionally, high PKM2 expression has been reported in malignant mesenchymal tumors and pancreatic cancer (PC), where its expression levels were closely linked to overall survival and progression-free survival outcomes in clinical cohorts (23). Collectively, these findings establish PKM2 as a consistently overexpressed factor across a broad spectrum of human malignancies, underscoring its pivotal role in tumor progression. The widespread dysregulation of PKM2 across diverse cancer types is further illustrated in **Figure 1**.

## 3 PKM2-mediated pathways in cancers

### 3.1 PKM2-mediated pathways in colorectal cancer

**Figure 2:**
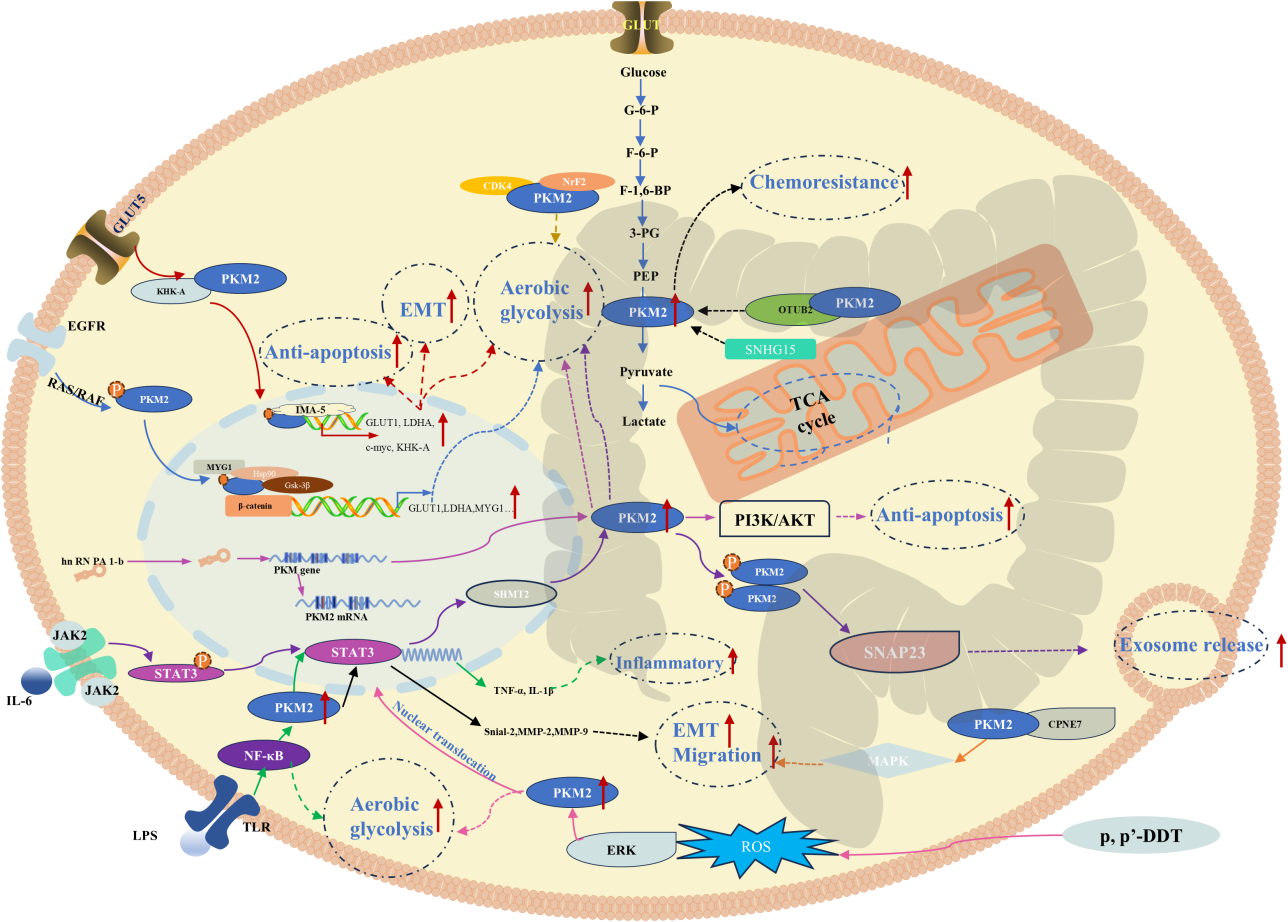
PKM2-mediated mechanisms and pathways in colorectal cancer.

CRC ranks as the third most prevalent malignancy globally and is associated with substantial mortality. Metabolic reprogramming, particularly the predominance of aerobic glycolysis, represents a hallmark of CRC pathophysiology. Given the pivotal role of PKM2 in aerobic glycolysis, extensive investigations continue to elucidate its mechanistic involvement in CRC progression. In colon cancer cells, melanocyte proliferating gene 1 (MYG1) facilitates the recruitment of the HSP90/GSK3β complex, thereby enhancing PKM2 stability and activity, which further amplifies aerobic glycolysis to drive tumor progression (24). Additionally, CDK4 interacts with PKM2, augmenting glycolytic flux and promoting malignant progression (25). The tumor-suppressive miR-142-3p, which exhibits reduced expression in CRC, directly targets the 3′-UTR of PKM2, modulating its PK-like activity (26). Clinically, loss-of-function mutations in APC are observed in 90% of patients with CRC, and these mutations contribute to aberrant activation of the β-catenin-PKM2 regulatory axis, thereby sustaining tumor growth through enhanced glycolytic metabolism (27). Moreover, PKM2 promotes aerobic glycolysis via the heterogeneous ribonucleoprotein A1-b (HnRNPA1-b)/PKM2 axis, while HnRNPA1-b enhances the Warburg effect by promoting PKM2 expression and amplifying the PI3K/AKT pathway (28). Beyond its function as a glycolysis rate-limiting enzyme in CRC cell proliferation, PKM2 is implicated in tumor progression through mechanisms involving altered adhesion and migration. The calcium-dependent phospholipid-binding protein CPNE7 interacts with PKM2 in CRC tissues, triggering MAPK signaling to accelerate tumor cell proliferation and migration (29). Furthermore, dimeric PKM2, endowed with protein kinase activity, promotes adhesion and facilitates metastatic dissemination by modulating STAT3-associated signaling pathways (30). Extracellular stimuli have been implicated in CRC progression through PKM2-mediated mechanisms. Upon fructose ingestion, Ketohexokinase-A (KHK-A) phosphorylates PKM2, inhibiting tetramer formation while concurrently promoting its nuclear translocation (31). This nuclear accumulation of PKM2 activates epithelial-mesenchymal transition (EMT) and aerobic glycolysis, enhancing migratory capacity and facilitating the loss of nest-dependent anti-mutagenic properties, particularly in CRC liver metastases. Furthermore, Nuclear PKM2 activates the transcription of c-myc regulatory genes GLUT1 and LDHA, thereby promoting the expression of the KHK-A subtype. Elevated KHK-A subsequently enhances cytoplasmic PKM2 phosphorylation, facilitating its nuclear translocation and thus establishing a positive feedback loop that intensifies metabolic reprogramming (31). Environmental organic pollutants, such as p,p′-DDT, have also been shown to upregulate PKM2 and promote its nuclear translocation *via* ROS-mediated ERK/PKM2 signaling, further potentiating glycolytic activity in CRC cells (32). In surgical patients, lipopolysaccharide (LPS) derived from intraoperative infections caused by Gram-negative bacteria activates the NF-κB pathway, leading to increased PKM2 binding to the STAT3 promoter. This interaction induces STAT3 pathway activation, facilitates STAT3 nuclear translocation, and drives the expression of TNF-α and IL-1β, exacerbating inflammation and contributing to CRC recurrence and metastasis (33). Moreover, STAT3 activation perpetuates disease progression through the STAT3/PKM2/SNAP23 signaling axis, leading to PKM2 phosphorylation, metabolic reprogramming *via* enhanced glycolysis, and increased exosome secretion by tumor cells (34). Tumor-derived exosomes transport a diverse array of bioactive molecules, among which the long noncoding RNA (lncRNA) HOTAIR binds to PKM2 in regulatory B cells, preventing its ubiquitin-mediated degradation. This interaction facilitates STAT3 activation and upregulates PD-L1 expression, thereby enhancing the immunosuppressive properties of CRC (35). Additionally, small nucleolar RNA host gene 15 (SNHG15) promotes CRC glycolysis by upregulating PKM2 expression and 5-FU resistance (36). Similarly, an OTU deubiquitinase (OTUB2), which exhibits high expression in CRC, directly inhibits PKM2 ubiquitination by blocking the interaction between PKM2 and its ubiquitin E3 ligase Parkin, thereby stabilizing its activity and ultimately driving resistance to chemotherapeutic agents (37). Conversely, the ubiquitin E3 ligase TRIM29 selectively targets PKM1 for degradation, indirectly increasing the relative abundance of PKM2 and shifting CRC metabolism toward aerobic glycolysis, thereby reinforcing malignancy (38). Based on accumulated evidence, the molecular pathways and regulatory mechanisms of PKM2 in CRC progression have been systematically delineated to construct a comprehensive PKM2-mediated signaling network in CRC (**Figure 2**). These findings underscore the potential of targeting PKM2-associated pathways as a promising therapeutic strategy for CRC treatment.

### 3.2 PKM2-mediated pathways in liver cancer

Liver cancer ranks as the fourth leading cause of cancer-related mortality worldwide, with HCC being the most prevalent and highly aggressive subtype (39, 40). PKM2 has been identified as a critical regulator in HCC progression, with clinical analyses of extensive HCC tissue samples revealing a strong correlation between PKM2 expression levels, tumor aggressiveness, and immune cell infiltration (41). Notably, elevated PKM2 expression has been linked to poor responsiveness to transarterial chemoembolization (TACE) in over 40% of patients with intermediate-stage HCC, contributing to chemoresistance and reduced survival (42). Beyond its role as a metabolic enzyme, PKM2 functions as a key regulatory protein facilitating HCC progression by driving aerobic glycolysis. Tumor-derived endogenous miR-624 accelerates HCC cell proliferation through PKM2 proteome modulation (43). Additionally, Bone Morphogenetic Protein 4 (BMP4) directly binds to the PKM promoter *via* SMAD5, upregulating PKM2 expression and enhancing glycolytic flux, thereby promoting glucose metabolism reprogramming and tumor progression (44). The deubiquitinase FAM188B stabilizes PKM2 by facilitating hnRNPA1(a key protein involved in RNA metabolism) deubiquitination, activating the hnRNPA1/PKM2 axis to upregulate aerobic glycolysis to accelerate HCC proliferation (45). PKM2 also promotes HCC metastasis *via* the JAK/STAT3 signaling pathway while concurrently suppressing autophagy by promoting autophagosome formations, further driving malignant progression (46). Epigenetic modifications of PKM2 contribute to HCC aggressiveness. Circular RNA HULC enhances PKM2 methylation, facilitating its involvement in autophagosome formation through CARM1 expression and driving malignant differentiation of HCC stem cells (47). Moreover, HCC cells elevate PKM2 expression by preventing its degradation, as ubiquitin-specific protease 35 (USP35) stabilizes PKM2 through deubiquitination, promoting aerobic glycolysis and tumor progression (48). Beyond its metabolic function, PKM2 acts as a transcriptional regulator in HCC. Histone deacetylase 8 (HDAC8) interacts with the PKM2 protein to perform deacetylation modification at position K62, reducing its cytoplasmic catalytic activity in glycolysis; however, this modification simultaneously facilitates PKM2 nuclear translocation, where it interacts with β-catenin to drive CCND1 transcription, disrupting cell cycle control and exacerbating tumor progression (49). Similarly, the intracellular lncRNA HClnc1 interacts with PKM2 to prevent its degradation, reinforcing aerobic glycolysis and activating PKM2-STAT3 signaling, which promotes angiogenesis, proliferation, chemoresistance, and anti-apoptotic mechanisms in HCC (50). Targeting PKM2 presents a potential therapeutic strategy, as Shikonin (SHK), which is isolated from *Lithospermum erythrorhizon*, has been shown to suppress PKM2 activity and glycolysis in HCC cells. However, in the refractory HCC cell line HCCLM3, SHK paradoxically induces PKM2 nuclear translocation, upregulating glycolysis-related gene transcription and metabolic activity (51). Additionally, high PKM2 expression in intrahepatic cholangiocarcinoma cells enhances resistance to gemcitabine by competitive inhibition of dNTP biosynthesis stimulates gemcitabine incorporation into DNA (52). In summary, PKM2 serves as a key oncogenic driver in HCC, particularly in HCC, exerting its influence through multiple mechanisms. Beyond its role in regulating aerobic glycolysis, PKM2 integrates into diverse interlinked signaling networks and functions as a protein kinase to modulate gene transcription.

### 3.3 PKM2-mediated pathways in breast cancer

Breast cancer (BC) remains a leading contributor to the female disease burden, exhibiting particularly high incidence rates in high-income countries (53). The malignancy of BC is significantly influenced by the expression profiles of estrogen receptor (ER), progesterone receptor (PR), and human epidermal growth factor receptor 2 (HER2). While chemotherapy remains the primary treatment modality for non-triple-negative BC (non-TNBC), PKM2-mediated pathways have been increasingly implicated in the development of chemoresistance. USP46, which is highly expressed in BC cells, enhances the PKM2/PKM1 ratio *via* the USP46/PTBP1/PKM2 axis, promoting glycolysis and conferring resistance to tamoxifen (54). Additionally, in the TBX15/miR-152/KIF2C axis, domain-2 of KIF2C directly binds to PKM2, preventing its ubiquitin-mediated degradation and increasing PKM2 protein stability. This stabilization contributes to doxorubicin (DOX) resistance by modulating both autophagy and aerobic glycolysis (55). Beyond its metabolic function, PKM2 also exhibits protein kinase activity, participating in the PKM2-c-Myc-survivin cascade, which regulates BC cell proliferation, migration, and tamoxifen resistance (56). In refractory TNBC, PKM2 has been strongly associated with chemoresistance. The E3 ubiquitin ligase TRAF6 directly interacts with PKM2, promoting PKM2-mediated glycolysis and enhancing drug resistance, as demonstrated in both preclinical models and clinical tumor samples (57). Moreover, protein arginine methyltransferase-1 (PRMT1) reinforces TNBC chemoresistance by upregulating lipid biosynthesis during aerobic glycolysis (58). Within the nucleus, PKM2 interacts with the histone methyltransferase EZH2, repressing the expression of multiple target genes and consequently influencing TNBC cell lineage specification (59). Collectively, PKM2 functions as a central regulator of glycolysis and a protein kinase that drives BC chemoresistance through diverse molecular pathways, irrespective of tumor subtype or malignancy.

### 3.4 PKM2-mediated pathways in gastric cancer

Gastric cancer (GC) remains one of the most prevalent and lethal malignancies of the digestive system. PKM2 has been identified as a key regulator in GC progression, participating in multiple signaling pathways that drive aerobic glycolysis and tumor proliferation. In GC cells, Src homology 2 domain-containing phosphatase 2 (SHP2) directly interacts with specific sites on PKM2, activating its enzymatic function and exacerbating glycolytic metabolism and malignant transformation (60). Additionally, the significantly upregulated lncRNA VAL in GC competes for PKM2 binding, reducing the interaction between PKM2 and Parkin, thereby suppressing PKM2 ubiquitination and enhancing its enzymatic activity (61). Enolase 1 (ENO1), another key glycolytic enzyme in GC, directly interacts with PKM2, facilitating aerobic glycolysis, tumor cell proliferation, migration, and apoptosis resistance (62). Beyond direct protein-protein interactions, endogenous signaling pathways also regulate PKM2-mediated GC progression. The highly expressed lncRNA CCAT1 promotes PKM2 expression through the PTBP1/PKM2/glycolysis axis, thereby enhancing glycolytic flux and driving tumor progression (63). Similarly, circular RNA circATP2B1, which is overexpressed in GC, functions as a competing endogenous RNA (ceRNA), directly binding to miR-326-3p and miR-330-5p, which serve as PKM2 inhibitors. This interaction leads to PKM2 upregulation, further promoting glycolysis and tumorigenesis (64). Moreover, histone methyltransferase SETD1A, which is highly expressed in GC tissues, interacts with and co-activates HIF1α, thereby upregulating multiple glycolytic enzymes, including PKM2, to reinforce glycolytic metabolism and tumor progression (65). Collectively, the abundant expression of PKM2 in GC cells, along with its involvement in various signaling cascades and protein interactions, plays a central role in driving aerobic glycolysis and malignant progression. These findings underscore the therapeutic potential of targeting intracellular PKM2 as a promising strategy for GC treatment.

### 3.5 PKM2-mediated pathways in pancreatic cancer

PC, a highly aggressive malignancy of obscure etiology, is often referred to as the “king of cancers” due to its exceptionally poor prognosis and resistance to conventional therapies. Research on PKM2 in PC remains in its early stages, yet emerging evidence suggests its significant involvement in key oncogenic processes.

The voltage-gated calcium channel α2δ1, expressed on the surface of PC cells, facilitates calcium influx, activating CaMKIIδ, which sequentially phosphorylates PKM2. This phosphorylation event promotes PKM2 nuclear translocation, ultimately enhancing the stem-like properties of PC cells and exacerbating tumor malignancy (66). Additionally, protein tyrosine phosphatase 1B (PTP1B), a putative oncogene in PC, modulates the PKM2/AMPK/mTORC1 signaling axis, increasing PKM2-associated protein kinase activity and enhancing mTORC1 activation, thereby accelerating PC cell proliferation (67). Tumor-associated macrophage-derived TGF-β1 further exploits PKM2 nuclear translocation to upregulate PD-L1 expression, facilitating immune evasion in PC (68). Moreover, upstream stimulatory factor 2 (USF2) negatively regulates lipid peroxidation and ferroptosis in PC cells by modulating PKM2-mediated transcriptional activity in the nucleus, further promoting tumor progression (69). Although comprehensive mechanistic insights into PKM2’s role in PC remain limited, existing studies highlight its potential significance in tumor stemness, immune escape, lipid peroxidation, and ferroptosis—hallmarks that drive PC malignancy. These findings provide a scientific foundation for further exploration of PKM2 as a therapeutic target in PC.

### 3.6 PKM2-mediated pathways in head and neck malignancies

Glioblastoma multiforme (GBM) is the most prevalent malignancy of the central nervous system, characterized by rapid progression and resistance to conventional therapies. Analysis of PKM2 expression in 119 patients with GBM identified it as an independent prognostic factor, with elevated levels correlating with poor survival outcomes, particularly in patients undergoing radiotherapy. These findings suggest that PKM2 represents a promising therapeutic target for GBM (70). In GBM cells with high ALDH1A3 expression, enhanced PKM2 tetramerization following interaction with ALDH1A3 not only augments aerobic glycolysis but also facilitates the lactylation and nuclear translocation of XRCC1, thereby promoting DNA damage repair and conferring resistance to temozolomide (TMZ) and radiotherapy (71). Additionally, reduced expression of the RNA-binding protein ZCRB1 and circular RNA circHEATR5B leads to the accumulation of their binding partner JMJD5, which interacts with PKM2 to enhance the formation of its highly active tetrameric state, further reinforcing glycolytic metabolism in GBM cells (72). In the MBNL1/circNTRK2/PAX5 pathway, the transcription factor PAX5, which is highly expressed in GBM, directly binds to the PKM2 promoter, increasing its transcription and protein expression, thereby sustaining tumor glycolysis and progression (73). Moreover, GBM resistance to TMZ has been linked to PKM2, with hypoxic TMZ-resistant cells transmitting resistance to TMZ-sensitive cells through exosomal transfer of PKM2 (74). Beyond GBM, PKM2 has also been implicated in other head and neck malignancies. In head and neck squamous cell carcinoma (HNSCC), Enolase 2 (ENO2), a crucial glycolytic enzyme in cancer metabolic process, directly interacts with PKM2, preventing its degradation while enhancing its glycolytic activity. This interaction also mediates AKT phosphorylation and promotes PKM2 nuclear translocation, contributing to malignant progression through cell cycle dysregulation (75). Furthermore, the accumulation of lactate driven by high PKM2 expression suppresses NF-κB signaling and enhances immunosuppressive capacity by upregulating the expression of Galectin-9, a key immunosuppressive factor in HNSCC (76). In cytotrophoblastoma, the mesenchymal-epithelial transition factor (C-MET) promotes PKM2 nuclear translocation *via* ERK1/2 phosphorylation, where it interacts with histone H3 to upregulate CCND1 and c-Myc, ultimately driving tumor proliferation and growth (77). Although research on PKM2 in head and neck cancers remains limited, existing studies highlight its critical role in therapy resistance, tumor metabolism, and immune evasion. Whether in GBM, where PKM2 contributes to chemoresistance and radiotherapy failure, or in other head and neck malignancies, where it facilitates aerobic glycolysis and immune suppression, these findings have redirected research focus toward the diverse oncogenic functions of PKM2, reinforcing its potential as a therapeutic target.

### 3.7 PKM2-mediated pathways in genitourinary cancers

**Figure 3:**
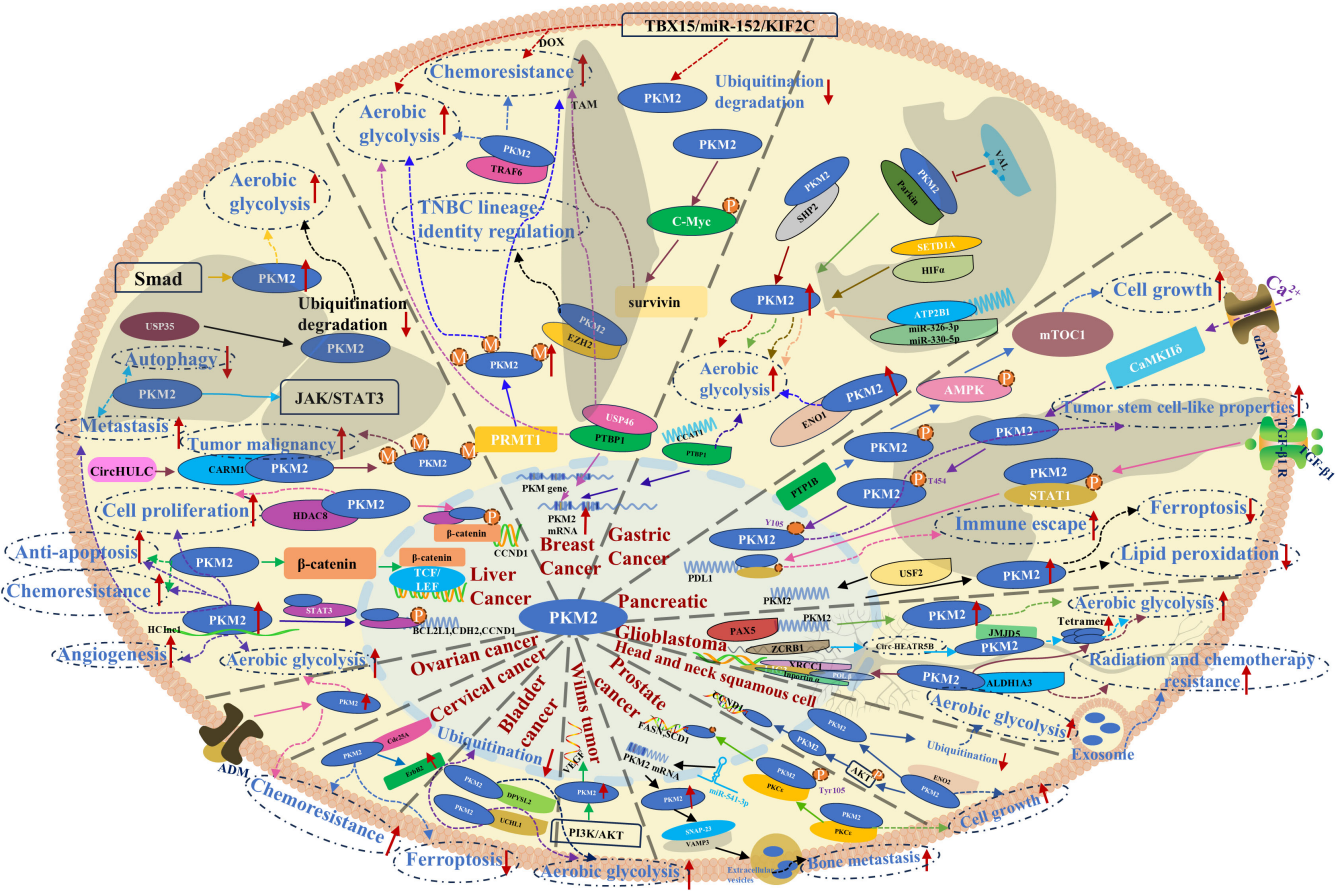
PKM2-mediated mechanisms and pathways in liver, breast, gastric, pancreatic, head and neck, and genitourinary system cancers.

Prostate cancer (PC) ranks among the most prevalent malignancies in men, with PKM2 playing a pivotal role in tumor progression and therapy resistance. Morphological and cytological analyses of PC cells have demonstrated that PKM2 is actively involved in the EMT, a process that facilitates lineage differentiation and contributes to resistance against antiandrogen therapies, ultimately driving tumor progression (78). In PC cells, PKM2 phosphorylation upon interaction with protein kinase C epsilon (PKCϵ) enhances its nuclear translocation, leading to the upregulation of oncogenes and the promotion of tumor proliferation (79). Bone metastases represent the primary cause of mortality in patients with metastatic PC. The lncRNA miR-541-3p has been identified as a key regulator of this process, facilitating PKM2 translational expression, which in turn promotes extracellular vesicle internalization and enhances metastatic potential in PC cells (80). Beyond PC, PKM2 has been implicated in other genitourinary malignancies. In bladder cancer, one of the most common urinary system malignancies, ubiquitin carboxy-terminal hydrolase L1 (UCHL1) directly binds to PKM2 and inhibits its ubiquitin-mediated degradation, leading to increased PKM2 protein levels and subsequently enhancing aerobic glycolysis, metastasis, and invasive activity (81). Similarly, in nephroblastoma, platelet-derived growth factor receptor-β (PDGFR-β) activates PKM2 *via* the PI3K/AKT pathway, promoting glycolytic metabolism while simultaneously upregulating VEGF transcription to drive tumor angiogenesis (82). PKM2 also plays a critical role in malignancies that predominantly affect women. Cervical cancer, a leading cause of cancer-related mortality in women, exhibits increased sorafenib resistance due to the Cdc25A/PKM2/ErbB2 pathway, in which Cdc25A suppresses ferroptosis by dephosphorylating PKM2 and upregulating ErbB2 expression (83). In ovarian cancer, platinum-based drug sensitivity is a key determinant of therapeutic efficacy. Anexelekto (Axl), a member of the TYRO3-AXL-MER receptor tyrosine kinase family, induces cisplatin resistance by phosphorylating PKM2 at Y105, thereby reinforcing glycolysis-driven tumor survival (84). Additionally, adrenomedullin has been found to promote aerobic glycolysis and cisplatin resistance in ovarian cancer by significantly upregulating PKM2 protein levels (85). Collectively, these findings underscore PKM2’s central role in tumor metabolism, therapy resistance, and metastatic progression across multiple cancer types. A comprehensive visualization of PKM2-mediated mechanisms in cancers of the liver, breast, stomach, pancreas, head and neck, and genitourinary system is presented in **Figure 3**.

## 4 Targeting PKM2 in tumors

### 4.1 Targeting PKM2 in colorectal cancer

The primary treatment modalities for CRC include surgery, chemotherapy, radiotherapy, and targeted therapy. In recent years, increasing attention has been directed toward targeted therapies, particularly those aimed at PKM2, which has emerged as a critical regulator of CRC metabolic reprogramming. Strategies targeting PKM2 at the protein or gene level have become a major focus in anti-CRC research.

Direct inhibition of PKM2 disrupts its PK activity, thereby suppressing CRC proliferation. TEPP-46, a small-molecule inhibitor, prevents PKM2 nuclear accumulation and promotes its tetramerization, ultimately inhibiting EMT and aerobic glycolysis in CRC cells (31). Similarly, the boronic acid-based compound 6C mitigates aerobic glycolysis and CRC progression by stabilizing the dimeric interface of PKM2 while facilitating its transition into a tetrameric conformation with reduced aerobic glycolytic activity (86). To enhance the efficacy of PKM2-targeting agents, nanoparticle-based delivery systems have been explored. SHK-loaded colloidal mesoporous silica nanoparticles selectively deliver inhibitors to CRC cells, effectively suppressing PKM2 activity and aerobic glycolysis, thereby inhibiting tumor proliferation (87). Similarly, SHK-loaded and hyaluronic acid-modified mesoporous polydopamine (MPDA) nanoparticles reprogram CRC metabolism by targeting PKM2, reversing EMT, and suppressing colorectal liver metastasis (88). Betulinic acid-loaded nanoliposomes have also been shown to inhibit PKM2-mediated aerobic glycolysis, disrupting CRC metabolic pathways to enhance anti-tumor activity (89). Beyond direct PKM2 inhibition, targeting PKM2-associated signaling pathways offers an alternative therapeutic strategy. miR-206 regulates the miR-206/hnRNPA1/PKM2 axis, inducing a PKM2-to-PKM1 isoform switch, which suppresses PKM2 expression and attenuates CRC aerobic glycolysis and proliferation (90). Additionally, miR-490-3p directly binds to hnRNPA1-b and modulates the miR-490-3p/hnRNPA1-b/PKM2 axis, promoting CRC apoptosis by enhancing the Warburg effect *via* the PI3K/AKT pathway (28). The lncRNA LiNC01852 modulates the TRIM72/SRSF5/PKM2 signaling axis, indirectly downregulating PKM2 expression through a multi-stage regulatory mechanism, thereby suppressing CRC cell proliferation and chemoresistance (91). Additionally, small molecules have been identified as indirect modulators of PKM2 expression. The CD36-Glypican 4 interaction inhibits β-catenin/c-Myc signaling, downregulating PKM2 expression and impairing glycolysis in CRC cells (92). N-acetyl-L-cysteine prevents PKM2 nuclear translocation and aerobic glycolysis induced by the environmental pollutant p,p’-DDT by inhibiting ERK1/2 activation and PKM2 upregulation (32). The synthetic compound diethyldithiocarbamate-copper complex suppresses CRC progression by promoting PKM2 ubiquitination and degradation through inhibition of the miR-16-5p and miR-15b-5p/ALDH1A3/PKM2 axis (93). Furthermore, NPD10084, as confirmed by the cellular thermal shift assay in colon cancer cells, disrupts PKM2 interactions with β-catenin and STAT3, thereby inhibiting downstream oncogenic signaling and suppressing CRC proliferation (94). In duodenal cancer cells, the indole-3-carbinol derivative OSU-A9 reduces nuclear pTyr105-PKM2 levels and promotes apoptosis by inhibiting ROS generation (95). Given concerns regarding chemical toxicity, nutritional interventions targeting PKM2 in CRC have gained traction. The dietary flavonoid kaempferol enhances miR-326 expression, directly targeting the 3’-UTR of PKM2 to inhibit glycolysis, thereby reversing 5-FU resistance in CRC cells (96). Kaempferol also inhibits CRC growth by promoting miR-339-5p expression, modulating the miR-339-5p-hnRNPA1/PTBP1-PKM2 axis to suppress PKM2 expression while upregulating PKM1 (97). Another dietary factor, apigenin, binds to the K433 site of PKM2, thereby impairing aerobic glycolysis and suppressing CRC cell proliferation (98). Additionally, ferulic acid and p-coumaric acid, derived from cereal bran, inhibit the lncRNA 495810/PKM2 axis, thereby suppressing glycolysis in CRC cells (99). Synergistic targeting of PKM2 through combinatorial approaches has also been explored. The co-administration of PKM2-siRNA and oxaliplatin exhibits enhanced anti-CRC efficacy (100). Additionally, SHK enhances the therapeutic effect of PD-1 blockade by modulating the SHK-PKM2-ROS-Hsp70 axis, thereby improving immune responses in CRC (101).

### 4.2 Targeting PKM2 in liver cancer

Characterized by its dual function as a classical aerobic glycolytic enzyme and a non-metabolic protein kinase-like entity in HCC cells, PKM2 has emerged as a critical therapeutic target, with numerous bioactive molecules demonstrating efficacy in suppressing HCC progression. Tumor-specific knockdown of PKM2 in a primary HCC model effectively reversed the Warburg effect and inhibited tumorigenesis in a genotype-dependent manner (102). The downregulation of PKM2 observed in sublethal heat treatment experiments, alongside tumor inhibition data, underscores its pivotal role in phase separation kinetics and pyroptotic pathways (103). ARHGAP24, an endogenous Rho-GTPase-activating protein in tumor cells, directly interacts with WWP1 and PKM2 to obstruct β-catenin signaling, thereby suppressing HCC cell proliferation and invasion (104). Additionally, the natural compound Chinese poplar propolis has been shown to attenuate aerobic glycolysis in HCC cells by reducing PKM2 protein levels *in vitro (* 105). Beyond monotherapies targeting PKM2, combination regimens designed to enhance anti-HCC efficacy through synergistic PKM2 inhibition have gained increasing attention. SHK suppresses PKM2 expression to disrupt aerobic glycolysis in hepatocellular carcinoma cells, thereby enhancing the therapeutic efficacy of sorafenib. Furthermore, nuclear PKM2 inhibition also downregulates CCND1 to block the cell cycle and exert antiproliferative effects (106). However, SHK also facilitates PKM2 nuclear translocation, activating Nrf2-Bcl2-associated athanogene 3 (BAG3) signaling to confer anti-apoptotic effects, a process counteracted by the addition of a BAG3 inhibitor, which enhances SHK-induced apoptosis (107). Furthermore, Canagliflozin suppresses aerobic glycolysis in HCC cells by targeting PKM2 expression and promoting the formation of the PKM2-c-MYC complex. Furthermore, this complex formation promotes the ubiquitination and degradation of c-MYC while reducing expression of the key enzyme GLS1 in glutamine metabolism. This impairs glutamine utilization, inducing intracellular glutamine starvation and subsequent ferroptosis, thereby sensitizing HCC cells to cisplatin treatment (108). The natural coumarin analog osthole, in combination with radiotherapy, suppresses aerobic glycolysis and enhances HCC radiosensitivity by inhibiting the GSK-3β/AMPK/mTOR pathway and downregulating PKM2 protein expression (109). Additionally, indirect targeting of PKM2 or its associated signaling axis represents an alternative therapeutic strategy. Cannabinoid receptor-interacting protein 1 (CNRIP1) inhibits intrahepatic cholangiocarcinoma cell proliferation, invasion, and migration by modulating the CNRIP1/Parkin/PKM2 pathway, thereby enhancing Parkin-PKM2 interactions and facilitating PKM2 ubiquitination and degradation (110). The multi-kinase inhibitor cabozantinib exerts potent anti-HCC effects by suppressing c-MET and ERK activity, leading to reduced PKM2 expression and impaired tumor angiogenesis (111). Inhibition of intracellular cleavage and polyadenylation-specific factor 6 (CPSF6) also suppresses PKM2 expression, thereby reversing the Warburg effect and impeding HCC progression (112). The ginsenoside metabolite compound K disrupts glycolysis and induces apoptosis in HCC cells by downregulating PKM2 through inhibition of the AKT/mTOR/c-MYC signaling pathway (113). Furthermore, antisense oligonucleotides (cEt/DNA ASO) promote PKM splicing transition, facilitating PKM2-to-PKM1 conversion, thereby restoring PK activity, normalizing glucose metabolism, and inhibiting HCC cell proliferation (114). Notably, fluorescent nanoparticles composed of carbon dots and dihydroartemisinin supramolecules suppress aerobic glycolysis *via* PKM2 inhibition while concurrently targeting the AKT/mTOR pathway to promote apoptosis. Beyond their therapeutic potential, these nanoparticles also offer real-time visualization, presenting a promising dual-functional approach for both HCC diagnosis and treatment (115).

### 4.3 Targeting PKM2 in breast cancer

Research on PKM2-targeted therapy in BC has advanced considerably, with numerous compounds demonstrating anti-BC effects by modulating PKM2 dimer-tetramer conversion and nuclear translocation. A novel glycopeptide-based PKM2 nano-activator has been developed to selectively accumulate in tumor-enriched regions, suppressing aerobic glycolysis in BC cells by sequestering PKM2 tetramers and preventing dimeric nuclear translocation. This metabolic shift effectively inhibits BC cell proliferation and metastasis while enhancing chemosensitivity (116). Additionally, β-elemene has been shown to counteract BC metastasis by regulating PKM2 dimerization and nuclear translocation, thereby modulating aerobic glycolysis in BC cells (117). Overexpression of the ubiquitin ligase Tripartite Motif-Containing 35 inhibits aerobic glycolysis by promoting PKM2 ubiquitination, thereby facilitating tetramer-to-dimer conversion (118). Furthermore, a novel sulfonamide-dithiocarbamate compound, 8k, suppresses BC cell proliferation by inhibiting PKM2 nuclear translocation and its downstream signaling cascade (119). Chinese poplar propolis has also demonstrated anti-proliferative effects in BC cells within an inflammatory microenvironment by targeting and downregulating PKM2, a key glycolytic enzyme (120). Innovative therapeutic strategies have emerged, including a nano-formulation integrating SiPKM2 with photothermal therapeutic materials, which precisely targets BC cells to disrupt PKM2-mediated aerobic glycolysis while synergistically potentiating photothermal ablation efficacy (121). Beyond direct PKM2 inhibition, several compounds exert antitumor effects by targeting upstream regulators of PKM2-associated signaling pathways. Pimozide, an antipsychotic agent, inhibits aerobic glycolysis and BC cell proliferation by suppressing the PI3K/AKT/MDM2 pathway, thereby upregulating p53 and downregulating PKM2 expression (122). Similarly, valproic acid impairs BC progression by inhibiting aerobic glycolysis through suppression of the HDAC1/ERK1/2/PKM2 axis (123). Additionally, cryptotanshinone, a bioactive component from traditional Chinese medicine, inhibits BC cell proliferation, migration, and invasion *via* downregulation of PKM2 through the PKM2/β-catenin signaling pathway (124).

TNBC, the most aggressive BC subtype, is characterized by poor prognosis due to its low chemotherapy sensitivity and lack of predictive biomarkers or targeted therapies. However, significant progress has been made in PKM2-targeted approaches against TNBC. Inhibition of immunoglobulin-like transcript 4 (ILT4) significantly downregulates AKT-mTOR-mediated PKM2 overexpression, thereby suppressing aerobic glycolysis and impairing TNBC cell proliferation, migration, and invasion (125). Additionally, a manganese dioxide-coated metal-organic framework-based nanomedicine has been designed to enhance the stability and targeted delivery of the gene-silencing drug SiPKM2, effectively suppressing tumor glycolysis and exerting potent anti-TNBC effects *in vivo (* 126). Combination strategies have also gained attention, with TEPP-46 demonstrated to synergize with CDK inhibitors by selectively binding PKM2^pS37^ and reducing its nuclear translocation, thereby amplifying anti-TNBC proliferation and invasion effects (127). Moreover, diindolylmethane (DIM), a dietary compound derived from cruciferous vegetables, not only reduces PKM2 expression to counteract aerobic glycolysis in TNBC cells but also enhances the efficacy of the anticancer agent Centchroman in TNBC treatment (128).

### 4.4 Targeting PKM2 in gastric cancer

Research on PKM2-targeted therapy in GC remains limited. DNA polymerase gamma has been identified as a binding partner of PKM2, modulating Tyr105 phosphorylation to suppress aerobic glycolysis and inhibit GC cell proliferation (129). Additionally, the traditional Chinese medicine formula Modified Jianpi Yangzheng Decoction (mJPYZ) has been shown to downregulate the PI3K/AKT/mTOR axis, promoting apoptosis while simultaneously reducing exosomal PKM2 secretion in GC (130). Further investigation revealed that mJPYZ also suppresses GC cell growth and EMT by inhibiting PKM2-dependent glycolysis through the PKM2/HIF-1α signaling pathway (131). Beyond direct PKM2 inhibition, indirect regulatory mechanisms also play a pivotal role. OSU-A9 increases intracellular ROS levels in GC cells, triggering apoptosis while concurrently reducing nuclear pTyr105-PKM2 expression (95). Despite the scarcity of research in this area, current findings underscore PKM2’s pivotal role in regulating key tumorigenic processes in GC, including exosomal PKM2 delivery, metabolic reprogramming, and apoptosis, highlighting its potential as a therapeutic target.

### 4.5 Targeting PKM2 in glioblastoma

GBM, the most lethal intracranial malignancy, presents significant therapeutic challenges, particularly in overcoming radioresistance following surgical resection. Notably, TEPP-46 has been shown to directly activate intracellular PKM2 in GBM cells, enhancing radiosensitivity without inducing cytotoxicity in normal human astrocytes (132). In parallel, advancements have been made in chemotherapy strategies targeting PKM2 in GBM. A supra-indication study on the sedative drug chlorpromazine revealed that it selectively binds PKM2 tetramers in GBM cells, inhibiting aerobic glycolysis and suppressing malignant progression, while exerting minimal effects on non-cancerous neuroepithelial cells (133). Additionally, the combination of PKM2 inhibitors (SHK+compound 3K) markedly enhances late apoptosis in U87MG glioma cells, demonstrating a potent anti-GBM effect (13). Moreover, the natural cytokine isopentenyladenosine inhibits the β/NF-κB pathway and downregulates PKM2 expression, thereby suppressing aerobic glycolysis in GBM cells (134). Collectively, these findings emphasize the therapeutic and adjuvant potential of PKM2-targeted interventions in gliomas, offering promising avenues for future clinical applications.

### 4.6 Targeting PKM2 in genitourinary tumors

Renal cell carcinoma (RCC), a highly aggressive malignancy of the urinary tract, exhibits significant chemoresistance, directly impacting patient prognosis. A novel nanoparticle drug, the PKM2 allosteric converter, has been shown to induce PKM2 tetramerization in RCC cells, thereby suppressing aerobic glycolysis. Reduced PKM2 dimerization concomitantly diminishes nuclear translocation, restoring cancer cell sensitivity to first-line chemotherapeutic agents (135). Similarly, a glycopeptide-based PKM2 nano-activator has demonstrated efficacy in PC by promoting PKM2 tetramer formation and preventing dimeric nuclear translocation, ultimately impairing PC cell proliferation, chemoresistance, and metastatic potential (116). Additionally, in the context of castration-resistant PC, the tanshinone IIA analog TB3 has been identified as an effective therapeutic agent, inhibiting the degradation of androgen receptor (AR), concurrently reducing AR nuclear translocation and thereby affecting AR-mediated transcription pathways of key ARE-containing genes, ultimately downregulating PKM2 expression by targeting this AR/PKM2 axis to suppress aerobic glycolysis (136). Further studies indicate that, Xihuang Pills (XHP), a traditional Chinese medicine compound tablet formulation, suppress PKM2 protein expression, significantly reducing aerobic glycolysis in chemoresistant PC cells (137).

PKM2 has also emerged as a critical metabolic biomarker in female reproductive malignancies, though targeted therapeutic strategies remain limited. Cryptotanshinone directly binds to PKM2, inhibiting its expression in ovarian cancer cells, thereby suppressing both aerobic glycolysis and oxidative phosphorylation (OXPHOS), leading to cell growth inhibition and apoptosis induction (138). Additionally, compound 3K has been shown to selectively suppress PKM2 expression in ovarian cancer cells, significantly impairing glycolytic capacity and inducing autophagy (139). Combination therapies targeting PKM2 have also demonstrated potential, with SHK significantly enhances the antitumor efficacy of olaparib, a poly (ADP-ribose) polymerase inhibitor that acts by blocking the homologous recombination pathway. The anti-tumor efficacy achieved by disrupting the homologous recombination pathway operates primarily through two mechanisms: firstly, SHK itself induces increased intracellular reactive oxygen species, leading to DNA double-strand breaks; secondly, SHK directly inhibits PKM2, thereby amplifying the effects of olaparib in inducing γH2AX upregulation, ATM phosphorylation activation, and BRCA1 downregulation (140). These findings underscore the therapeutic significance of targeting PKM2 in genitourinary and gynecologic malignancies, highlighting its potential role in overcoming aerobic glycolysis-mediated chemoresistance.

### 4.7 Targeting PKM2 in other tumors

Beyond the extensive body of research on PKM2-targeted therapies in common systemic malignancies, a smaller yet highly innovative subset of studies has explored cutting-edge therapeutic strategies targeting PKM2 in less frequently studied cancers. In the context of esophageal squamous cell carcinoma (ESCC), photodynamic therapy (PDT) has been shown to directly inhibit PKM2, thereby activating the PKM2/caspase-8/caspase-3/GSDME axis. This pathway suppresses aerobic glycolysis and promotes programmed cell death in ESCC cells, suggesting that PDT-mediated ESCC treatment is fundamentally linked to PKM2 inhibition (141, 142). Additionally, the small molecule compound 8 has been identified as a modulator of mitochondrial function, disrupting the PKM2-VDAC3 (a regulator of ferroptosis) interaction to inhibit tumor growth *in vivo* while concurrently inducing ferroptosis, highlighting a novel connection between PKM2 and iron-dependent cell death (143). PKM2 has also been implicated in melanoma, where it is highly expressed. Benserazide, a known inhibitor, directly binds to PKM2, suppressing aerobic glycolysis while upregulating OXPHOS, thereby inhibiting melanoma proliferation, including in BRAFi-resistant cells (22). Furthermore, HA344 has been found to covalently bind to PKM2, effectively counteracting melanoma drug resistance by inhibiting tumor cell glycolysis (144). A Metabolic Reprogramming Immunosurveillance Activation Nanomedicine (MRIAN) has been engineered to enhance immunosurveillance against leukemia cells. Upon degradation, MRIAN suppresses PKM2 activity and lowers ROS levels, thereby disrupting the immunosuppressive properties of leukemia cells and inducing their differentiation into normal hematopoietic lineages (145).

**Figure 4:**
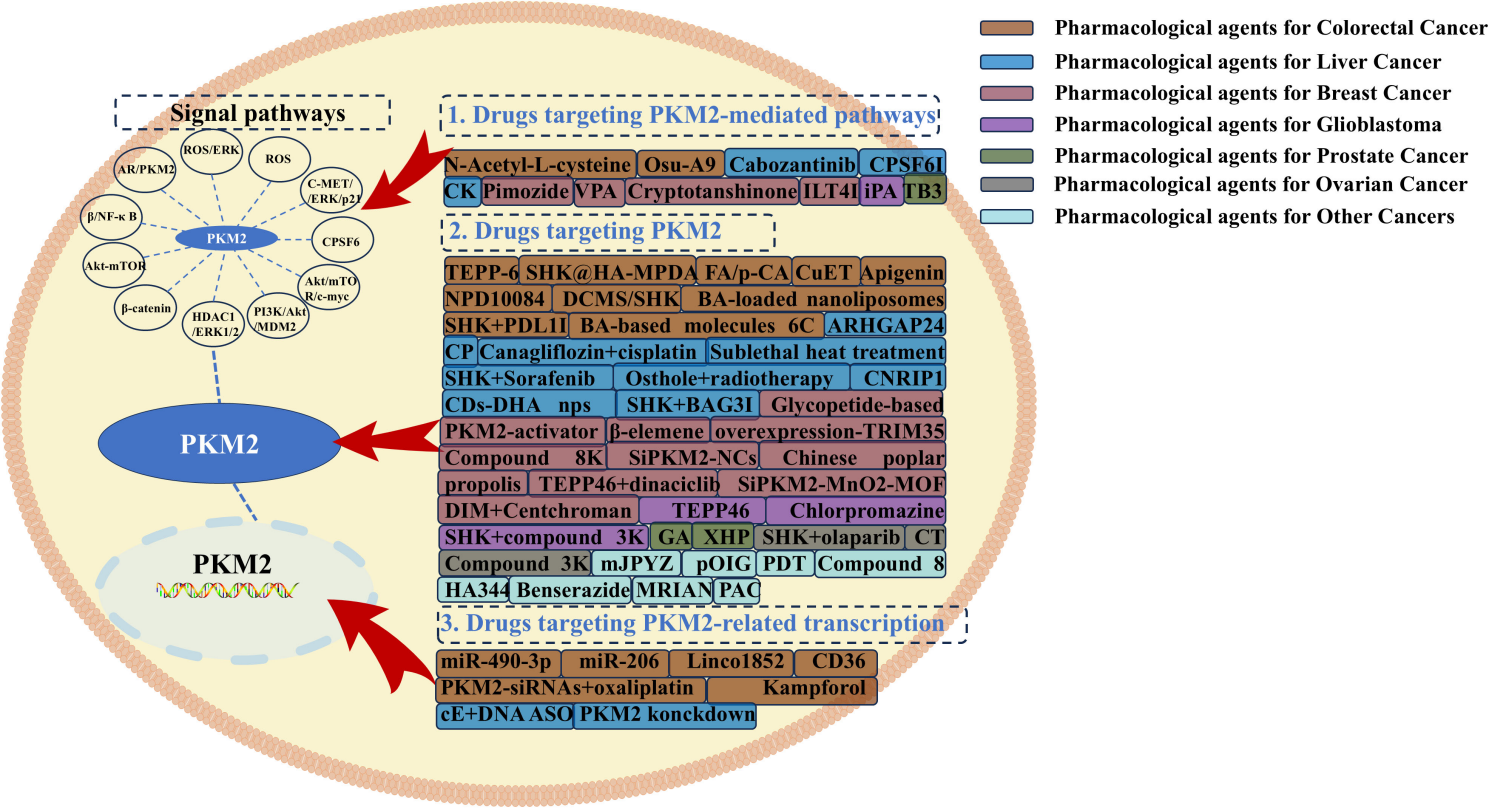
Therapeutic strategies targeting PKM2-mediated pathways, PKM2, and PKM2-mediated transcription in colorectal cancer, liver cancer, breast cancer, glioblastoma, prostate cancer, ovarian cancer, and other tumors.

These findings underscore the significant therapeutic potential of PKM2-targeted interventions across various malignancies. Given the extensive body of evidence supporting the efficacy of PKM2 inhibition in tumor therapy, a systematic summary has been compiled, categorizing recent advancements into three primary strategies: direct targeting of PKM2 protein, modulation of PKM2-associated nuclear transcripts, and indirect inhibition *via* regulatory signaling pathways that modulate PKM2 activity. A schematic representation of these therapeutic approaches is provided in **Figure 4**.

## 5 Conclusions and future perspectives

As the principal rate-limiting enzyme of aerobic glycolysis, PKM2 is aberrantly overexpressed in numerous malignancies, playing a critical role in tumor cell metabolism. However, accumulating evidence indicates that PKM2 exacerbates tumor progression beyond its metabolic function, contributing to oncogenesis through diverse non-metabolic mechanisms and signaling pathways. Moreover, PKM2 has been extensively studied as a tumor-associated protein kinase. The emergence of specific PKM2 inhibitors such as Shikonin (SHK) and Compound 3 has further reinforced the association between PKM2 and malignancy (51, 146, 147). In multiple human malignancies, including colorectal, hepatocellular, breast, gastric, and pancreatic cancers, PKM2 not only enhances aerobic glycolysis but also promotes tumor progression *via* alternative pathways. In CRC, aberrant PKM2 overexpression and nuclear translocation drive EMT, migration, immunosuppression, and inflammatory progression through non-metabolic mechanisms. Similarly, PKM2-mediated β-catenin signaling in HCC confers resistance to apoptosis and chemotherapy in a non-metabolic manner. In BC, particularly triple-negative subtypes, PKM2 regulates tumor phenotypes, modulates autophagy, and contributes to doxorubicin resistance. Furthermore, elevated PKM2 nuclear translocation in genitourinary tumors has been identified as a key factor in chemoresistance and metastatic dissemination. Building on these mechanistic insights, the emergence of PKM2-targeted therapies has significantly reshaped the current oncological treatment landscape. More intriguingly, the series of malignant effects induced by PKM2 translocation—such as cell cycle arrest, DNA damage repair, EMT progression, and the activation of associated transcription—may all be regulated by its function as an intranuclear RNA-binding protein. Given the expanding research on PKM2-mediated oncogenic pathways and targeted therapeutics, a systematic collation of recent findings is presented in **Table 1**. Despite advancements, investigations into PKM2-driven pathways remain insufficient, with numerous tumor-promoting effects arising from PKM2 interactions with other proteins yet to be elucidated. FurthHER2 exploration of these molecular mechanisms holds considerable promise. Therapeutic strategies targeting PKM2 have demonstrated notable efficacy, with existing approaches achieving anti-tumor effects through direct inhibition of PKM2 protein, modulation of PKM2-associated nuclear transcription, and indirect suppression of PKM2 activity *via* aerobic glycolysis, immunosuppression, and resistance to chemotherapy and radiotherapy. These approaches have already yielded substantial breakthroughs. More innovatively, multimodal strategies, including combinatorial drug regimens, radio- and chemotherapy integration, thermal ablation, and PKM2-targeted nanotherapeutics, have demonstrated significant therapeutic potential.

**Table 1:** Summary of PKM2-Mediated mechanisms and therapeutic strategies in tumours.

Tumor types	PKM2-mediated mechanisms or pathways	Functions	PKM2-targeted treatments	Validation level	Reference
Colorectal cancer	MYG1 recruits HSP90/GSK3β complex, ↑PKM2 stability, ↑PKM2 activity	↑Aerobic glycolysis and CRC progression		Cell, Clinical samples, In vivo (animals)	(24)
Colorectal cancer	OTUB2 ↓PKM2 degradation, ↑PKM2 activity	↑Aerobic glycolysis, Proliferation, Migration and Chemotherapy drug resistance		Cell, in vivo (animals)	(37)
Colorectal cancer	STAT3/PKM2/SNAP23	↑Aerobic glycolysis, Exosome release and CRC progression		cell, in vivo (animals)	(34)
Colorectal cancer	CDK4-PKM2	↑Aerobic glycolysis and CRC progression		Cell, Clinical samples, In vivo (animals)	(25)
Colorectal cancer	TRIM29 ↓PKM1, ↑PKM2	↑CRC malignant phenotype		Cell, Clinical samples, In vivo (animals)	(38)
Colorectal cancer	SNHG15 ↑PKM2 expression	↑Aerobic glycolysis and 5-FU resistance capacity		Cell	(36)
Colorectal cancer	3 ′-UTR of miR-142-3p-PKM2, ↑PKM2 activity	↑Aerobic glycolysis, Invasion and Migration		Clinical samples, In vivo (animals)	(26)
Colorectal cancer	Regulatory B cell: HOTAIR-PKM2, ↓PKM2 degradation, STAT3 activation, PDL1(+)	↑Immune suppression		Cell, in vivo (animals)	(35)
Colorectal cancer	APC loss: β-catenin-PKM2, ↑PKM2 activity	↑Aerobic glycolysis and Proliferation		Cell, Clinical samples, In vivo (animals)	(27)
Colorectal cancer	LPS ↑NF-κB pathway: ↑PKM2/STAT3, ↑PKM2 nuclear translocation, ↑TNF-α and IL-1β	↑Inflammatory progression, CRC recurrence and Metastasis		Cell	(33)
Colorectal cancer	CPNE7-PKM2, ↑MAPK pathway	↑Proliferation and Migration		Cell, Clinical samples, In vivo (animals)	(29)
Colorectal cancer	PKM2/STATT3	↑Migration and Adhesion		Cell	(30)
Colorectal cancer	hnRNPA1-b ↑PKM2 expression, PI3K/AKT pathway	↑Aerobic glycolysis, Proliferation and Anti-apoptosis	miR-490-3p (Targeting hnRNPA1-b, an upstream gene of PKM2 transcription)	Cell, Clinical samples	(28)
Colorectal cancer	p,p'-DDT ↑ROS-mediated ERK/PKM2, ↑PKM2 expression and nuclear translocation	↑Aerobic glycolysis	N-Acetyl-L-cysteine (Targeting ROS, an upstream signalling molecule of ERK/PKM2)	Cell	(32)
Colorectal cancer	KHK-A ↑PKM2 nuclear translocation, ↓PKM2 tetramer	↑Aerobic glycolysis, EMT, Migration and Anti-apoptosis	TEPP-46 (Targeted inhibition of PKM2 phosphorylation and translocation)	Cell, Clinical samples, In vivo (animals)	(31)
Colorectal cancer	TRIM72/SRSF5/PKM2, ↓PKM2 expression	↑Proliferation and Chemotherapy drug resistance	lncRNA LiNC01852 (Targeted inhibition of SRSF5-mediated PKM2 alternative splicing)	Cell, Clinical samples, In vivo (animals)	(91)
Colorectal cancer	CD36-GPC4-β-catenin-c-myc signal axis, ↓PKM2 gene	↓Aerobic glycolysis	CD36 (Targeting the proteasome-dependent ubiquitination of GPC4 upstream of PKM2)	Cell, Clinical samples, In vivo (animals)	(92)
Colorectal cancer	↓PKM2, ↓lactate, ↓TGF-β signaling	↓Aerobic glycolysis, EMT and CRLM	SHK@HA-MPDA (Targeting PKM2 for direct inhibition)	Cell, in vivo (animals)	(88)
Colorectal cancer	miR-16-5p and15b-5p/ALDH1A3/PKM2 axis, ↑PKM2 ubiquitination degradation	↓Aerobic glycolysis	CuET (Targeting ALDH1A3, an interacting protein of PKM2)	Clinical samples, In vivo (animals)	(93)
Colorectal cancer	lncRNA 495810/PKM2 axis, ↓PKM2	↓Aerobic glycolysis	FA, p-CA (Targeting lncRNA 495810, inhibiting the lncRNA 495810/PKM2 axis.)	Cell, In vivo (animals)	(99)
Colorectal cancer	miR-326-hnRNPA1/A2/PTBP1-PKM2 axis, ↓PKM2 expression	↓Aerobic glycolysis, Reversing 5-Fu resistance	Kaempferol (Up-regulating miR-326 , directly inhibits PKM2)	Cell	(96)
Colorectal cancer	miR-339-5p-hnRNPA1/PTBP1-PKM2 axis, ↓PKM2 expression	↓Aerobic glycolysis and Proliferation	Kaempferol (Up-regulating miR-339-5p , inhibiting hnRNPA1/PTBP1-PKM2 axis)	Cell	(97)
Colorectal cancer	K433 site of PKM2	↓Aerobic glycolysis	apigenin (Targeting PKM2 for direct inhibition)	Cell, In vivo (animals)	(98)
Colorectal cancer	↓PKM2	↓CRC	PKM2-siRNAs+oxaliplatin	Cell	(100)
Colorectal cancer	↓PKM2-β-catenin /STAT3	↓CRC proliferation	NPD10084 (Targeting the interaction between PKM2 and either β-catenin or STAT3)	Cell, In vivo (animals)	(94)
Colorectal cancer	↓PKM2	↓Aerobic glycolysis and Proliferation	DCMS/SHK (Targeting PKM2 for direct inhibition)	Cell	(87)
Colorectal cancer	↑ROS, ↓pTyr105-PKM2	↑Apoptosis	OSU-A9 (Generating ROS subsequently downregulates nuclear PKM2)	Cell, In vivo (animals)	(95)
Colorectal cancer	↓PKM2	↓Aerobic glycolysis	BA-loaded nanoliposomes (Inhibiting PKM2)	Cell	(89)
Colorectal cancer	Shikonin-PKM2-ROS-Hsp70' axis	↓CRC	SHK+PD1 blocker (Targeting PKM2 for direct inhibition)	Cell, In vivo (animals)	(101)
Colorectal cancer	miR-206/hnRNPA1/PKM2 axis, ↓PKM2 expression	↓Aerobic glycolysis and Proliferation	miR-206 (Targeting hnRNPA1 , inhibiting PKM2 expression)	Cell	(90)
Colorectal cancer	↓PKM2 dimer, ↑PKM2 tetramer	↓Aerobic glycolysis and Proliferation	boronic acid-based molecules 6C (Targeting PKM2 Dimerisation)	Cell	(86)
Liver cancer	HDAC8/PKM2, ↑PKM2 nuclear translocation, ↑CCND1 transcription	↓Aerobic glycolysis, ↑Proliferation		Cell, Clinical samples, In vivo (animals)	(49)
Liver cancer	PKM2 proteome	↑Proliferation		Cell, In vivo (animals)	(43)
Liver cancer	SHK ↑PKM2 nuclear translocation	↑Aerobic glycolysis		Cell	(51)
Liver cancer	USP35 ↓PKM2 deubiquitination degradation	↑Aerobic glycolysis, Proliferation, Migration and Invasion		Cell, Clinical samples, In vivo (animals)	(48)
Liver cancer	CircHULC ↑PKM2 methylation modification	↑Hepatocellular carcinoma stem cell malignancy		Cell, In vivo (animals)	(47)
Liver cancer	Expression of PKM2	Clinical features and Immune cell abundance correlation		Clinical samples	(41)
Liver cancer	PKM2-mediated JAK/STAT3 pathway	↑HCC metastasis, ↓Autophagy		Cell, Clinical samples	(46)
Liver cancer	PKM2↑	↑Drug resistance, ↓Survival		Cell, Clinical samples	(42)
Liver cancer	hnRNPA1/PKM2 axis	↑HCC progression		Cell, In vivo (animals)	(45)
Liver cancer	Smad signal pathway, ↑PKM2	↑Aerobic glycolysis		Cell, Clinical samples	(44)
Liver cancer	HClnc1-PKM2, ↓PKM2 degradation, ↑PKM2-STAT3 signaling	↑Aerobic glycolysis, Proliferation, angiogenesis, 5-Fu and OXA resistance and Anti-apoptosis		Cell, Clinical samples, In vivo (animals)	(50)
Liver cancer	ARHGAP24/WWP1/PKM2/β-catenin axis, ↑PKM2 degradation	↓Proliferation and Invasion	Overexpressed ARHGAP24 (Targeting PKM2 to promote degradation)	Clinical samples	(104)
Liver cancer	c-MET/ERK/p21/PKM2 cascade, ↓PKM2	↓HCC	Cabozantinib (Suppressing c-MET and ERK activity, downregulating PKM2)	In vivo (animals)	(111)
Liver cancer	↑PKM shear conversion, ↑PKM1, ↓PKM2	↑Normal glycolysis, ↓HCC progression	cEt/DNA ASO (Inducing selective expression of PKM1)	Cell, In vivo (animals)	(114)
Liver cancer	Reversing the Warburg effect	↓Primary hepatocarcinogenesis	PKM2 Knockdown	In vivo (animals)	(102)
Liver cancer	↓CPSF6, ↓PKM2	↓Aerobic glycolysis	CPSF6 inhibition (Reducing PKM2 Expression)	Cell, Clinical samples, In vivo (animals)	(112)
Liver cancer	PKM2-C-MYC complex, ↓PKM2	↓Aerobic glycolysis, ↓Cisplatin resistance	Canagliflozin (Targeting PKM2)	Cell, Clinical samples, In vivo (animals)	(108)
Liver cancer	Phase separation dynamics and pyroptotic pathways, ↓PKM2	↓HCC	Sublethal Heat Treatment	Cell	(103)
Liver cancer	↓PKM2	↓Aerobic glycolysis	CP (Inhibiting PKM2 protein levels)	Cell	(105)
Liver cancer	↓PKM2, ↓BCL-2, ↓CyclinD1	↓Aerobic glycolysis and Proliferation, ↑Apoptosis and Orafenib efficacy	SHK+Orafenib (Targeting PKM2 for direct inhibition)	Cell, In vivo (animals)	(106)
Liver cancer	GSK-3β/AMPK/mTOR pathway, ↓PKM2	↓Aerobic glycolysis, ↑Radiosensitization	osthole+irradiation (Inhibiting PKM2 protein levels)	Cell	(109)
Liver cancer	↓PKM2, ↓Akt/mTOR signaling pathway	↓Aerobic glycolysis, ↑Apoptosis and Imageability	CDs-DHA nanoparticles (Inhibiting PKM2 protein levels)	Cell, In vivo (animals)	(115)
Liver cancer	↓AKT/mTOR/c-Myc signal axis, ↓PKM2	↓Aerobic glycolysis, ↑Apoptosis	compound K (Targeting the PKM2 Upstream AKT/mTOR/c-Myc Signalling Pathway)	Cell	(113)
Liver cancer	SHK ↓PKM2 activity,↑PKM2 nuclear translocation, BAG3I ↓Nrf2-BAG3	↑Apoptosis	SHK+BAG3I (Targeting PKM2 for direct inhibition)	Cell, In vivo (animals)	(107)
Intrahepatic cholangiocarcinoma	↑PKM2, β-catenin signaling cascades	↑Anti-apoptosis and gemcitabine resistance		Cell, Clinical samples, In vivo (animals)	(52)
Intrahepatic cholangiocarcinoma	CNRIP1/Parkin/PKM2 pathway, ↑PKM2 ubiquitination degradation	↓Proliferation, Invasion and Migration	Overexpressed CNRIP1 (Activate the E3 ubiquitin ligase Parkin to degrade PKM2)	Cell, Clinical samples, In vivo (animals)	(110)
Breast Cancer	TBX15/miR-152/KIF2C axis, ↓PKM2 ubiquitination degradation	↑Aerobic glycolysis, Autophagy and DOX resistance		Cell, In vivo (animals)	(55)
Breast Cancer	PKM2-c-Myc-survivin cascade	↑Proliferation, Migration and Tamoxifen resistance		Cell	(56)
Breast Cancer	USP46/PTBP1/PKM2 axis, ↑PKM2/PKM1	↑Aerobic glycolysis and Tamoxifen resistance		Cell, Clinical samples, In vivo (animals)	(54)
Breast Cancer	PKM2-TRAF6	↑Aerobic glycolysis and Chemotherapy drug resistancee		Cell, Clinical samples, In vivo (animals)	(57)
Breast Cancer	Binding PKM2 tetramer, ↓PKM2 dimerization nuclear translocation	↓Aerobic glycolysis, Proliferation, Translocation and Drug resistance	Glycopeptide-based PKM2 nano-activator (Inducing PKM2 Tetramerisation)	Cell, In vivo (animals)	(116)
Breast Cancer	PI3K/Akt/MDM2 signaling pathway, ↑p53, ↓PKM2	↓Aerobic glycolysis and Proliferation	Pimozide (Blocking the PI3K/Akt/MDM2 signalling pathway upstream of PKM2 , downregulating PKM2 expression)	Cell, In vivo (animals)	(122)
Breast Cancer	↓PKM2 dimer, ↑PKM2 tetramer	↓Aerobic glycolysis and Proliferation	Overexpressed TRIM35 (Regulating the ubiquitin modification of PKM2 to promote its conversion from dimers to tetramers)	Cell, Clinical samples, In vivo (animals)	(118)
Breast Cancer	↓PKM2 nuclear translocation, ↓PKM2 downstream pathway	↓Proliferation	Sulfonamide-dithiocarbamate compound 8k (Reducing the nuclear localisation of PKM2 and blocking its downstream signalling pathways)	Cell	(119)
Breast Cancer	↓PKM2 dimer, ↑PKM2 tetramer, ↓PKM2 nuclear translocation	↓Aerobic glycolysis and Metastasis	β-elemene (Blocking the conversion between dimeric and tetrameric forms of PKM2)	Cell	(117)
Breast Cancer	↓HDAC1/ERK1/2/PKM2	↓Aerobic glycolysis and Proliferation	VPA (Downregulating PKM2 expression)	Cell, In vivo (animals)	(123)
Breast Cancer	PKM2/β-catenin axis, ↓PKM2	↓Proliferation, Migration and Invasion	Cryptotanshinone (Inhibiting PKM2/β-catenin signalling)	Cell	(124)
Breast Cancer	↓PKM2	↓Aerobic glycolysis, ↑PTT efficacy	SiPKM2-NCs	Cell, In vivo (animals)	(121)
Breast Cancer	↓PKM2	↓Aerobic glycolysis and Proliferation	Chinese Poplar Propolis (Downregulating PKM2 expression)	Cell	(120)
Breast Cancer TNBC	PRMT1 ↑PKM2 methylation	↑Aerobic glycolysis and Chemotherapy drug resistancee		Cell, Clinical samples, In vivo (animals)	(58)
Breast Cancer TNBC	PKM2-EZH2	Management of TNBC lineage identity		Cell	(59)
Breast Cancer TNBC	↓Phosphorylation of PKM2 S37 site, ↓PKM2 nuclear translocation	↓Proliferation and Invasion	TEPP-46+dinaciclib (Targeting PKM2 reduces nuclear localisation)	Cell, Clinical samples, In vivo (animals)	(127)
Breast Cancer TNBC	↓AKT-mTOR, ↓PKM2	↓Aerobic glycolysis, Proliferation and Metastasis	ILT4I (Downregulating PKM2 expression)	Cell, In vivo (animals)	(125)
Breast Cancer TNBC	↓PKM2	↓Aerobic glycolysis	SiPKM2-MnO2-MOF	Cell, In vivo (animals)	(126)
Breast Cancer TNBC	↓PKM2	↓Aerobic glycolysis and Proliferation	DIM+Centchroman (Downregulating PKM2 expression)	Cell, In vivo (animals)	(128)
Gastric cancer	CCAT1/PTBP1/PKM2/glycolysis pathway, ↑PKM2	↑Aerobic glycolysis and GC progression		Cell, Clinical samples, In vivo (animals)	(63)
Gastric cancer	VAL-PKM2/Parkin, ↓PKM2 ubiquitination, ↑PKM2 enzyme activity	↑Aerobic glycolysis and GC progression		Cell, Clinical samples, In vivo (animals)	(61)
Gastric cancer	SETD1A-HIFα, ↑PKM2	↑Aerobic glycolysis and GC progression		Cell, Clinical samples, In vivo (animals)	(65)
Gastric cancer	circATP2B1-miR-326-3p/miR-330-5p, ↑PKM2	↑Aerobic glycolysis and GC progression		Cell, Clinical samples, In vivo (animals)	(64)
Gastric cancer	ENO1-PKM2, ↑PKM2	↑Aerobic glycolysis and GC progression		Cell	(62)
Gastric cancer	SHP2/PKM2/AMPK axis, ↑PKM2	↑Aerobic glycolysis and GC progression	SHP099 (Targeting SHP2 to inhibit its dephosphorylation and activation of PKM2)	Cell, Clinical samples, In vivo (animals)	(60)
Gastric cancer	↓PI3K/Akt/mTOR, ↓Exosomal PKM2	↓Malignant progression of gastric cancer-associated macrophages	mJPYZ (Downregulating PKM2expression)	Cell, In vivo (animals)	(130)
Gastric cancer	PKM2/HIF-1α signaling	↓Aerobic glycolysis, Proliferation and EMT	mJPYZ (Downregulating PKM2 expression)	Cell, In vivo (animals)	(131)
Gastric cancer	↓Activation of PKM2 site phosphorylation	↓Aerobic glycolysis and GC progression	PolG (Targeting PKM2 directly)	Cell, Clinical samples, In vivo (animals)	(129)
Gastric cancer	↑ROS, ↓pTyr105-PKM2	↑Apoptosis	OSU-A9 (Generating ROS subsequently downregulates nuclear PKM2)	Cell, In vivo (animals)	(95)
Pancreatic	PTP1B/PKM2/AMPK/mTOC	↑Cell growth		Cell, Clinical samples, In vivo (animals)	(67)
Pancreatic	α2δ1-PKM2, ↑PKM2 nuclear translocation	↑Tumor stem cell activity		Cell, Clinical samples, In vivo (animals)	(66)
Pancreatic	USF2/PKM2, ↑PKM2 transcription	↓Lipid peroxidation and iron-dead cell death		Cell	(69)
Pancreatic	TGF-β/PKM2-pSTAT1/PDL-1	↑Immune escape	TEPP46 (Targeting PKM2 reduces nuclear localisation)	Cell, Clinical samples, In vivo (animals)	(68)
Glioblastoma	MBNL1/circNTRK2/PAX5, ↑PKM2 transcription	↑Aerobic glycolysis		Cell, Clinical samples, In vivo (animals)	(73)
Glioblastoma	ZCRB1/circHEATR5B/HEATR5B-881aa/JMJD5/PKM2, ↑PKM2 tetramer	↑Aerobic glycolysis		Cell, Clinical samples, In vivo (animals)	(72)
Glioblastoma	Exosomal delivery of PKM2↑	↑TMZ Resistance		Cell	(74)
Head and neck cancer	ENO2-PKM2, ↑PKM2 activity and nuclear localization, ↓PKM2 protein degradation	↑Aerobic glycolysis and Cycle progression		Cell, Clinical samples, In vivo (animals)	(75)
Head and neck cancer	Lactic acid produced by PKM2 upregulation, ↓NF-κB signaling pathway, ↑Galectin-9	↑Immunosuppression and Malignant progression		Cell, Clinical samples, In vivo (animals)	(76)
Retinoblastoma	ERK 1/2/PKM2/histone H3 signalling pathway	↑Proliferation and Tumor growth		Cell, In vivo (animals)	(77)
Glioblastoma	ALDH1A3-PKM2, ↑PKM2 tetramer, ↑Emulsification and nuclear localization of XRCC1	↑Aerobic glycolysis and Radiotherapy tolerance	D34-919 (Targeting the ALDH1A3-PKM2 interaction)	Cell, Clinical samples, In vivo (animals)	(71)
Glioblastoma	Brain PKM2 activation	↑Radiotherapy sensitivity	TEPP46 (Targeting PKM2 reduces nuclear localisation)	Cell, In vivo (animals)	(132)
Glioblastoma	Chlorpromazine-PKM2 tetramer	↓Aerobic glycolysis	Chlorpromazine(Targeting PKM2 directly)	Cell	(133)
Glioblastoma	↓PKM2	↑Apoptosis and Autophagy	SHK+compound 3K(Targeting PKM2 directly)	Cell, Clinical samples	(13)
Glioblastoma	β/NF-κ B, ↓PKM2	↓Aerobic glycolysis	iPA(Downregulating PKM2 expression)	Cell, Clinical samples	(134)
Prostate cancer	PKM2 mediates lineage identity	↑Anti-androgen drug resistance and Tumor progression		Cell, Clinical samples, In vivo (animals)	(78)
Prostate cancer	PKCϵ-PKM2, ↑PKM2 nuclear translocation	↑De novo lipogenesis and Proliferation		Cell, In vivo (animals)	(79)
Prostate cancer	↑PKM2 expression, ↑extracellular vesicles	↑Bone metastasis		Cell, Clinical samples, In vivo (animals)	(80)
Prostate cancer	↑PKM2 tetramer, ↓PKM2 dimer nuclear translocation	↓Aerobic glycolysis, Proliferation, Chemoresistance and Metastasis	OGA(Activating PKM2 Tetramerisation)	Cell, In vivo (animals)	(116)
Prostate cancer	AR/PKM2 axis	↓Aerobic glycolysis	TB3(Targeting AR, regulating the AR/PKM2 axis)	Cell, In vivo (animals)	(136)
Prostate cancer	↓PKM2	↓Aerobic glycolysis	XHP (Downregulating PKM2 expression)	Cell, In vivo (animals)	(137)
Bladder cancer	DPYSL2-PKM2, ↑PKM2 activity	↑Malignant, Aerobic glycolysis and EMT		Cell, Clinical samples, In vivo (animals)	(146)
Bladder cancer	UCHL1-PKM2, ↓PKM2 ubiquitinated degradation, ↑PKM2 protein	↑Aerobic glycolysis, Proliferation, Metastasis and Invasion		Cell, Clinical samples	(81)
Cervix	Cdc25A/PKM2/ErbB2 pathway	↓Cellular iron death, ↑Sorafenib resistance		Cell, Clinical samples, In vivo (animals)	(83)
Ovarian cancer	ADM ↑PKM2 protein	↑Aerobic glycolysis, Proliferation and Platinum drug resistance		Cell	(85)
Ovarian cancer	PKM2-Y105 Phosphorylation	↑Aerobic glycolysis and Cisplatin resistance		Cell, Clinical samples, In vivo (animals)	(84)
Ovarian cancer	↓PKM2	↑Olaparib antitumor activity	SHK+ olaparib (SHK,Targeting PKM2 directly)	Cell, Clinical samples, In vivo (animals)	(140)
Ovarian cancer	CT-PKM2, ↓PKM2	↓Aerobic glycolysis, Oxidative phosphorylation and Proliferation	CT (Targeting PKM2 directly)	Cell, In vivo (animals)	(138)
Ovarian cancer	↓PKM2	↓Aerobic glycolysis, ↑Autophagy	Compound 3K (Targeting PKM2 directly)	Cell, Clinical samples, In vivo (animals)	(139)
Nephroblastoma	PI3K/AKT/PKM2 pathway, ↑PKM2	↑Aerobic glycolysis and Angiogenesis		Cell	(82)
Renal cancer	↑PKM2 tetramer, ↓PKM2 dimer nuclear translocation	↓Aerobic glycolysis, ↑Chemosensitivity	PAC (Inducing PKM2 Tetramerisation)	Cell, Clinical samples, In vivo (animals)	(135)
Melanoma	↑HA344-PKM2	↓Aerobic glycolysis, ↑Chemosensitivity	HA344 (Targeting PKM2 directly)	Cell, In vivo (animals)	(144)
Melanoma	↑Benserazide-PKM2	↓Aerobic glycolysis, ↑Chemosensitivity	Benserazide (Targeting PKM2 directly)	Cell	(22)
Leukaemia	↓PKM2 activity	↓Immunosuppression, ↑Normal cell differentiation	MRIAN (Suppressing PKM2 activity)	Cell, In vivo (animals)	(145)
Esophageal cancer	PKM2/caspase-8/caspase-3/GSDME axis	↓Aerobic glycolysis, ↑Pyrolysis	PDT (Targeting PKM2 activates pyroptosis)	Cell, Clinical samples, In vivo (animals)	(141)
Esophageal cancer	↓PKM2	↓Aerobic glycolysis	PDT (Downregulating PKM2 expression)	Cell	(142)
Others	↓PKM2-VDAC3, ↑Cell iron death	↓Proliferation	Compound 8 ( Blocking the interaction between PKM2 and VDAC3 (a regulator of ferroptosis))	Cell	(143)

BA, betulinic acid; CP, Chinese Poplar Propolis; CRC, colorectal cancer; CRLM, colorectal cancer liver metastasis; DOX, doxorubicin; EMT, epithelial, mesenchymal transition;SHK, Shikonin; HCC, hepatocellular carcinoma; OXA, oxaliplatin; PDT, photodynamic therapy; PTT, photothermal therapy; TMZ, temozolomide; TNBC, triple, negative breast cancer; VPA, Valproic acid.

However, based on current findings, numerous challenges and shortcomings remain. In-depth exploration of the specific mechanisms of PKM2 as a nuclear transcription factor and RNA-binding protein. Many studies concerning targeted anti-tumor drugs for PKM2 have remained confined to the description of purely phenomenological results, without delving into more profound investigations of the underlying mechanisms and pathways. Future PKM2-targeted treatment strategies can be refined and expanded based on these emerging paradigms. However, whether targeting PKM2 directly or its mediated pathways, the inherent toxicity of therapeutic agents, their low selectivity for PKM2, and the costs associated with formulation development all pose challenges that currently constrain the clinical translation of PKM2-targeting drugs as therapeutic options. In conclusion, PKM2 serves as a key therapeutic target for malignant tumors due to its abnormal expression. Furthermore, utilizing the stage-specific expression of PKM2 throughout the entire tumor treatment cycle as a potential therapeutic efficacy biomarker represents a promising strategy. Both approaches collectively open highly prospective innovative pathways for cancer treatment. The continued development of PKM2-targeted agents is expected to improve patient outcomes across a broad spectrum of malignancies, positioning PKM2 inhibition as a transformative strategy in precision oncology.
